# Rare Case of Scapula Resection for Oligometastatic Colon Carcinoma to Bone

**DOI:** 10.1002/cnr2.70310

**Published:** 2025-09-01

**Authors:** Shaan Sadhwani, Jamie Henzes, Joshua Harman, Brian Omslaer, Emily Kelly, Lauren Zeitlinger

**Affiliations:** ^1^ Department of Orthopaedic Surgery UPMC Central PA Harrisburg Pennsylvania USA

**Keywords:** chemotherapy, colon cancer, radiation, resection, scapula

## Abstract

**Background:**

Colorectal carcinoma with isolated metastasis to the scapula is a rare occurrence. There is a paucity of accounts detailing experience with this unique scenario. We present a case of oligometastatic colon adenocarcinoma to the scapula with subsequent scapulectomy, in which the patient had eventual visceral disease recurrence yet still gained a palliative benefit from the procedure.

**Case:**

We detail the account of a patient with metastatic colorectal carcinoma to the right scapula and subsequent treatment for the scapular metastasis. The patient underwent successful total scapulectomy for metastatic colon adenocarcinoma with negative margins achieved. He had an uneventful postoperative course and was discharged home on day three. Upon follow‐up after total scapulectomy, the patient experienced significant symptom improvement and functional recovery.

**Conclusion:**

We believe a significant benefit to the patient's overall quality of life was provided by undergoing this procedure. Therefore, this option should remain part of the oncologic surgeon's armamentarium to offer a palliative option to patients with the goal of controlling pain and retaining function.

## 1 Introduction

Colorectal carcinoma (CRC) is the third most common cause of cancer in men and women in the United States [1, 2]. As survival improves for patients with CRC, the prevalence of bone metastasis and potential for metastases at previously uncommon sites continues to rise [3, 4]. While distant metastases from CRC are most frequently spread to the lung and liver, bony metastasis can occur in about 3%–11%, usually in conjunction with these visceral sites [5, 6, 7, 8]. Risk factors for bone metastasis include high‐grade tumors, lung and lymph node involvement [3]. The scapula remains a rare site of metastasis not only in CRC but of all types of metastatic tumors, with rates in the literature around 1%–4% [9]. Furthermore, the scapula as a singular site of bony metastasis is an exceedingly rare occurrence [10].

When encountered, scapular metastasis can have a wide range of possible treatment options from palliative radiation to forequarter amputation, resulting in a wide range of functional and prognostic outcomes for patients. One must remember that a functional limb is pivotal to a patient's independence, and therefore limb preservation should always be a goal of treatment [9]. In deciding on treatment options for these patients, the principles of limb‐sparing surgery should be considered. These include: local recurrence should be no greater and survival no worse than with amputation; the procedure or subsequent complications should not delay adjuvant therapy; reconstruction should be enduring; and function of the limb should be greater than or at least equal to that obtained by amputation [11].

Due to the rarity of this type of metastasis, there is a paucity of data regarding experiences or definitive guidelines for treatment that aims to find a balance between the functional and oncologic outcomes. We present a case of oligometastatic colon adenocarcinoma to the scapula with subsequent scapulectomy, in which the patient had eventual visceral disease recurrence yet still gained a palliative benefit from the procedure. In doing so, we elaborate on our perioperative considerations and surgical techniques to add to the paucity of literature currently available regarding this unique situation. The patient provided written informed consent for publication of case details and use of images.

## 2 Case History

**FIGURE 1:**
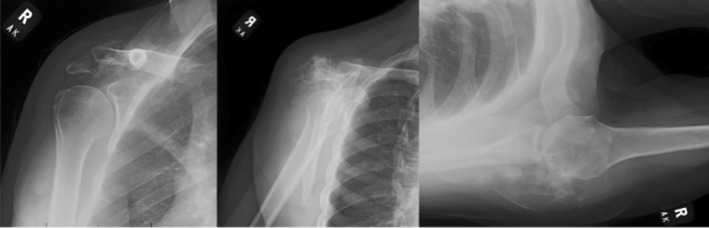
Anteroposterior, scapular‐Y, and axillary radiographs depicting a large heterogeneous mass arising from the right scapula with evident calcifications and bony destruction of the acromion and spine of the scapula which can be seen at the superior aspect of the shoulder joint.

A 48‐year‐old male who presented to a UPMC outpatient clinic and initially sought medical attention due to shoulder swelling of unknown duration in September of 2020. He was subsequently diagnosed with rectal cancer with metastatic disease to the liver and a large lytic lesion in the right acromion in the following weeks. Despite delays caused by the COVID‐19 pandemic, a confirmed diagnosis was made 3 months later. The patient initially underwent chemotherapy and was started on denosumab, which resulted in the resolution of the liver lesion (Table 1). He then underwent 15 fractures of radiation to the shoulder, followed by chemoradiation to the rectum, colectomy, and was placed on maintenance chemotherapy. He subsequently had the resolution of shoulder pain but the lesion itself persisted, and post‐radiation changes led to limited range of motion and decreased function, which affected his quality of life. He then presented to our clinic 3 years later, in which imaging showed an increase in scapular lesion size and recurrent shoulder pain, prompting orthopedic oncologic evaluation (Figure 1). Advanced imaging showed an expansile lesion arising from the posterior superior scapular spine with central necrosis and significant bony destruction.

**TABLE 1:** Summarized course of patient's diagnosis, treatment, procedures, and outcomes.

Time	Events
September 2020	Diagnosed with rectal cancer with metastatic disease to the liver and a large lytic lesion in the right acromion
10/2020–4/2021	Started on chemotherapy and denosumab
5/2021–6/2022	Adjustments made to chemotherapy regimen but continued chemotherapy
6/2022–7/2022	R scapula radiation, total of 15 fractions
8/2022–10/2022	Chemoradiation to the rectum
12/2022	Colectomy
1/2023	Started on maintenance chemotherapy
6/2023	Return of R scapula pain. Only evidence of active disease in R scapula. Underwent R scapulectomy
11/2023	Mildly elevated CEA levels. CT/PET revealed new visceral metastasis to the liver and adrenals
12/2023	Restarted on chemotherapy
4/2025	No evidence of disease on most recent scans and continues taking maintenance chemotherapy

**FIGURE 2:**
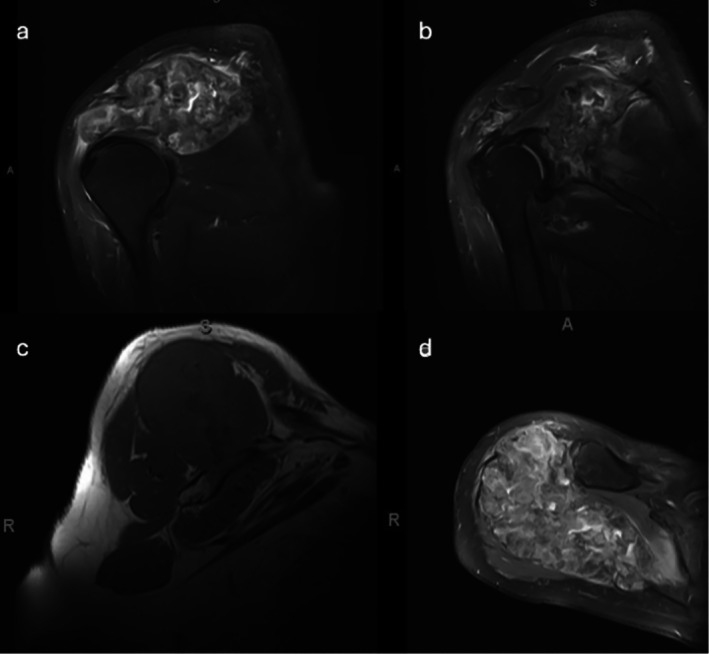
(a) Coronal T2 weighted MRI, (b) coronal T2 weighted MRI, (c) sagittal T1 MRI, and (d) axial T2 MRI of the right scapula all depicting a large heterogenous scapular mass with extensive soft tissue component and bony destruction. It also demonstrates lesion progression near the glenoid neck and in close proximity to the articular surface of the glenohumeral joint. T2 images also depict extensive inflammatory changes due to the reaction of the soft tissue as well as the resultant mass effect from the tumor.

**FIGURE 3:**
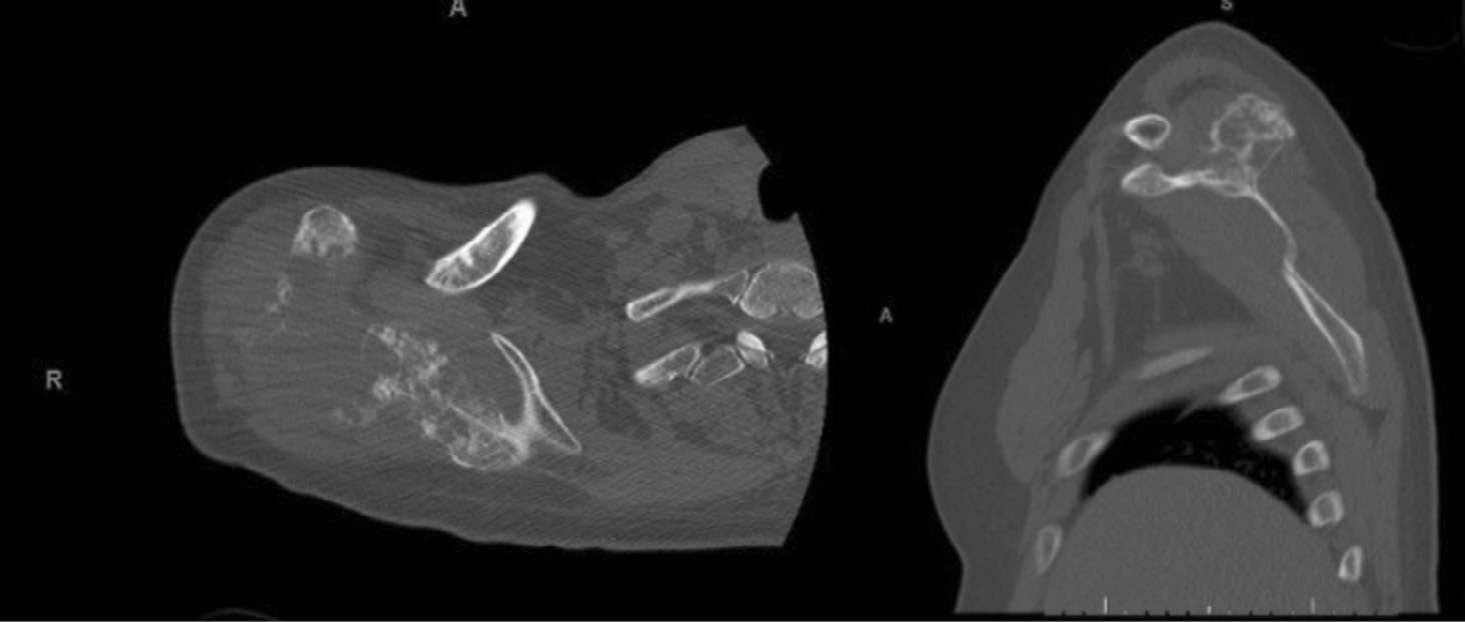
Coronal and sagittal CT scan showing a large right scapular mass with extensive bony destruction seen through the evident cortical erosion. This finding is extremely remarkable with the loss of much of the acromion and scapular spine cortical bone on the coronal CT.

**FIGURE 4:**
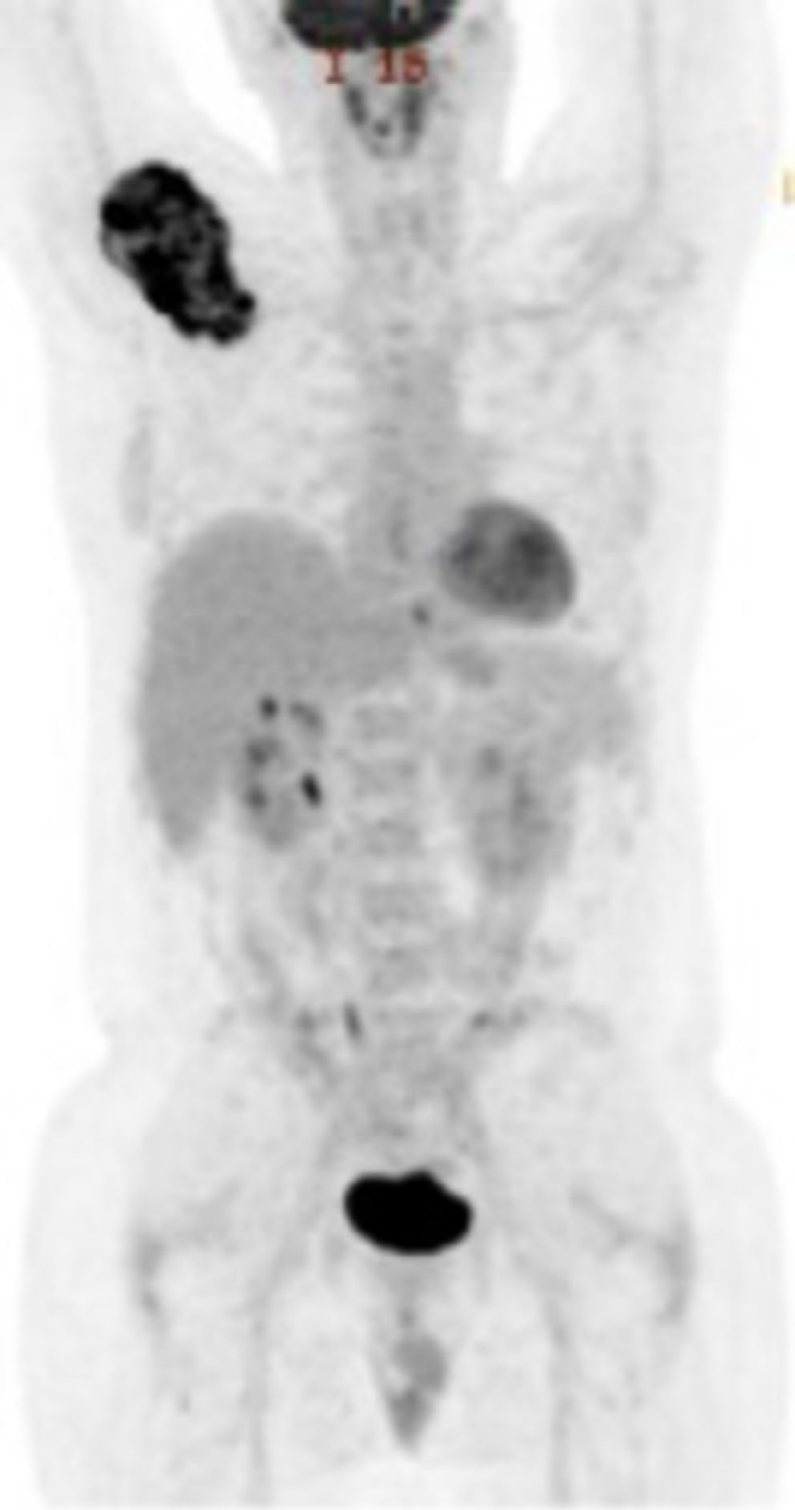
Whole body PET/CT scan depicting oligometastatic disease to the right scapula with no other sites of evident metastasis.

Once obtained, computed tomography (CT) and magnetic resonance imaging (MRI) demonstrated increased enhancement and destruction (Figures 2 and 3). Extensive discussion regarding possible surgical intervention for the scapular lesion was had, as he was deemed disease‐free otherwise by medical/oncological/surgical teams. Repeat PET scan demonstrated substantial interval enlargement of the destructive lytic lesion now involving the majority of the scapula, including the glenoid neck, with no other sites of metastasis identified (Figure 4). Advanced discussions were had regarding surgical treatment options, as the pain and discomfort had vastly diminished the patient's quality of life. It was understood that there would be a high risk of morbidity in attempting this type of surgery, and that obtaining negative margins was more significant than attempting glenoid preservation. It was also acknowledged that while wide resection could render him with no evidence of disease, it would not necessarily render a curative surgery. After all treatment options were discussed with the patient and family, the decision to procedure with total scapulectomy was made in an attempt to treat his pain and functional limitations. Maintenance chemotherapy was held 1 week prior to surgery.

### 2.1 Operative Technique

**FIGURE 5:**
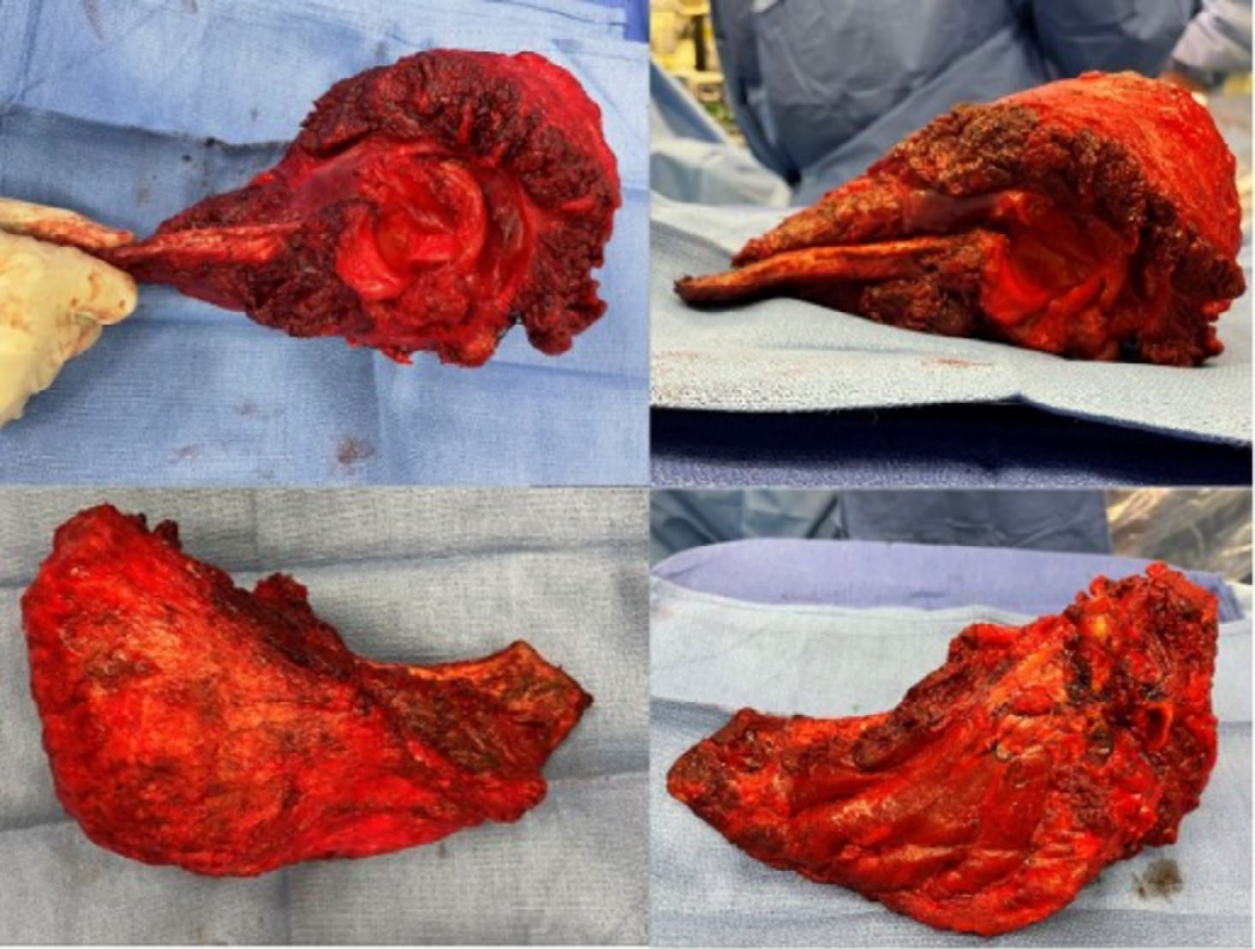
Clinical photos of the excised right scapula with evident tumor burden.

The patient was placed in the lateral decubitus position on the operating table. An extensile utilitarian approach to the scapula was utilized and extended anteriorly via the deltopectoral interval. This combination allowed full exposure of both the anterior and posterior scapular structures and musculature attachments. Full‐thickness fascial‐cutaneous flaps were elevated, with careful subcutaneous dissection to areas overlying the acromion due to post‐radiation changes and tumor abutment of the deep fascia. The deltoid was released laterally from the acromion due to soft tissue extent in the lateral deltoid and extended both anteriorly over the humerus and posteriorly through the platysma and trapezius to the medial border of the scapula to perform scapular excision (Figure 5). Following scapular excision, suspension arthroplasty of the humerus was performed using the remaining tendons of supraspinatus, infraspinatus, conjoint tendon, and mersilene tape. Bone tunnels through the distal clavicle were prepared, and the tape was passed through and tied once appropriate tensioning of the humerus was achieved. The humeral head was then covered with the remaining deltoid, trapezius, pec major, and teres minor to achieve both static and dynamic suspension. A layered closure was performed over a drain to ensure there was minimal fluid accumulation within the created dead space.

### 2.2 Postoperative Course

The patient underwent successful total scapulectomy for metastatic colon adenocarcinoma with negative margins achieved. He had an uneventful postoperative course and discharged home on day three. Upon follow‐up after total scapulectomy, the patient experienced significant symptom improvement and functional recovery. Postoperatively, carcinoembryonic antigen (CEA) tumor markers were followed. He was found to have a substantial reduction to 10.3 ng/mL from his maximum level of 418 ng/mL preoperatively (local lab reference range: 0.0–7.2 ng/mL). These levels continued to decreased to 7.2 ng/mL until 7 months after his procedure, in which an increase to 11.1 ng/mL was found. Most recently, his CEA level was 6.4 ng/mL at 8 months from his procedure. However, approximately 5 months postoperatively, the patient was found to have elevated CEA levels and advanced imaging demonstrating disease progression to the liver and left adrenal gland. He underwent palliative chemotherapy with a positive response, with tumor markers trending down towards baseline. At the most recent follow‐up appointment at the 6‐month postoperative mark, he continues to endorse resolution of shoulder pain and is satisfied with the functional outcome. Functionally, the patient is able to perform limited active abduction and flexion of his right shoulder, allowing him to touch his own face. In turn, this allows him to perform basic tasks essential to daily life, such as eating and hygiene. He remains neurovascularly intact distally with full range of motion at the elbow, forearm, wrist, and hand. He has been able to return to work full time without limitations in a desk job and can even drive a manual transmission vehicle safely. A summarized timeline of events is provided in Table 1.

## 3 Discussion

Given that CRC is one of the most common cancers in the United States, understanding the common metastatic locations is a crucial aspect of its management. While metastasis to the liver and lungs is most common, bone metastasis occurs in only 3%–11% of cases, in which isolated skeletal metastasis accounts for 1%–6% [7, 8, 10, 12]. Furthermore, bone metastasis in CRC is reported to be most common in the spine, followed by the pelvis and long bones [13, 14]. Risk factors associated with bony metastasis include primary rectal locations, lung and lymph node metastasis, and high serum CEA [8]. In our patient, he did have a primary rectal location with liver metastasis and elevated CEA. In any case, bone metastasis confers a poor prognosis, and therefore its early detection is crucial [2, 8, 13]. Indicators of poor prognosis in CRC patients with bone metastasis include multiple sites, high levels of CEA, osteolytic lesions, hypercalcemia, and pathologic fracture [14]. Interestingly, it has been found that while multiple sites of bony metastasis found late in the disease course carries a median survival of 5–7 months, isolated bone metastases carry a significantly better prognosis with superior 5‐year survival to those with bone and visceral metastasis [14]. In our patient's preoperative investigation, he did present with high CEA levels. However, these levels substantially decreased after surgery. Furthermore, the imaging results obtained from the radiographic investigation indicated isolated bony metastasis, which aided in prognostic decision making and resection planning.

We present a case that falls into an exceedingly rare subset of CRC patients with bone metastasis, in which our patient was found to have oligometastatic disease to his scapula (after resolution of liver metastasis after chemotherapeutic treatment). Despite the volume of literature available about metastatic colon cancer, there remains a paucity of publications regarding the management of a singular lesion to the scapula. Onesti et al. [10] describe the first known occurrence in a 2011 case report reporting a patient who underwent forequarter amputation due to a singular 12 cm × 15 cm metastatic lesion to the scapula following a Stage IIb colon cancer diagnosis. In this case, the forequarter amputation procedure resulted in a favorable outcome for the patient. However, other reports show relatively poor overall 5‐year survival with significant physiologic and psychological stress placed on patients who undergo forequarter amputation [15]. Given the extensive and functionally altering nature of a forequarter amputation, surgeons must have other limb‐sparing options in their armamentarium. In a study by Kiss et al. [5], 91 cases of limb‐preserving procedures were evaluated, in which 27 of those patients had metastatic disease and 13 patients underwent total scapulectomy. Even though active shoulder movements were limited postoperatively in these patients, the lack of pain and preserved fine motor function of the hand were enough to provide good patient satisfaction [5]. These results mirror our patient, who reported excellent post‐operative results regarding pain control and functional outcome.

Unfortunately for our patient, 5 months after resection he was found to have recurrent metastasis to his liver and was restarted on chemotherapy. However, to date at 6 months follow‐up, there has been no local recurrence after his resection. Interestingly, Krishnan et al. [4] examined local recurrence of bony metastasis after surgery in 301 cases and found that surgical margins and primary cancer type were independent risk factors of local recurrence, with colon cancer having the highest rate at 31%. Furthermore, it has been found that local recurrence is not associated with disease burden, implying that local control after surgery for bone metastasis is affected more by surgical margins than systemic factors. While this rate may be high, the surgeon must weigh the potential to achieve negative margins and the functional benefit the patient will gain from resection when considering operative options. In our case, we believed that we would be able to achieve a negative margin resection and help the patient in terms of the pain and functional deficit he had from both the lesion and post‐radiation changes present. Additionally, he demonstrated rapid progression of the tumor in terms of size and pain after radiation therapy. When weighing this against the potential surgical morbidity and change for both local and systemic recurrence, the patient and our team felt that at the very least, the palliative benefits outweighed the risk given the promising prognostic data for patients with isolated bony metastasis without visceral involvement in CRC [14]. With the rapid progression, there was concern that continued morbidity would ensue should a non‐operative approach be taken. Although this patient had recurrence of visceral metastasis, this report adds to the current literature that is lacking regarding management of an isolated scapular metastatic lesion associated with CRC and the perioperative and surgical considerations surrounding this situation. While these decisions can be challenging, it should always be remembered that a multidisciplinary approach to these patients should be taken to ensure optimal patient outcomes [11].

## 4 Conclusion

We present a patient who underwent total scapulectomy for metastatic colon adenocarcinoma with negative margins achieved at the time of the initial procedure. Postoperatively, he experienced significant symptom improvement and functional recovery. Despite this patient experiencing metastatic disease recurrence to the liver 5 months postoperatively, we believe a significant benefit to the patient's' overall quality of life was provided by undergoing this procedure. Therefore, this option should remain part of the oncologic surgeon's armamentarium to offer a palliative option to patients with the goal of controlling pain and retaining function.

## Author Contributions

**Shaan Sadhwani:** writing – original draft, project administration, investigation. **Jamie Henzes:** writing – original draft, investigation. **Joshua Harman:** writing – original draft, investigation. **Brian Omslaer:** writing – review and editing. **Emily Kelly:** writing – review and editing. **Lauren Zeitlinger:** supervision, conceptualization.

## Conflicts of Interest

The authors declare no conflicts of interest.

## Data Availability

The data that support the findings of this study are available on request from the corresponding author. The data are not publicly available due to privacy or ethical restrictions.
